# Dialysis*“Graham on Liquid Diffusion applied to Analysis,” *Royal Society’s Transactions* for 1861, Part I. “Redwood on Dialysis,” *Pharmaceutical Journal* for April, 1862. Daubeny’s Lectures on Agricultural Chemistry,” *Gardeners’ Chronicle* for December 7 and 14, 1861.

**Published:** 1863-03

**Authors:** 


CHICAGO
MEDICAL JOURNAL.

VOL. VI.] MARCH, 1863. [No. 3.

### SELECTED.

### DIALYSIS.*

Human ingenuity is advancing the art of chemical analysis with rapid strides. It is becoming no easy matter to keep oneself at all *au niveau* with the discoveries in this department of science. While yet fascinated with the beauties and subtleties of Spectral Analysis, our attention is claimed for another analytical discovery, less beautiful it is true and less subtle, but susceptible of much wider application, yielding results of greater practical value, and therefore possessing more immediate interest to us as medical men. We allude to the discovery of Dialysis, which we owe to Mr. Graham, the present Master of the Mint. It may be fairly described as a kind of royal road or short cut, enabling us to arrive at analytical results previously unattainable, or attainable only by processes far more complicated, far more open to fallacy. Except in rare instances it employs no chemical reagents; it achieves its end merely by availing itself of certain physical properties inherent in the substances to be analyzed. Our readers may possibly welcome an account of the principles of

* "Graham on Liquid Diffusion applied to Analysis," *Royal Society's Transactions* for 1861, Part I. "Redwood on Dialysis," *Pharmaceutical Journal* for April, 1862. Daubeny's Lectures on Agricultural Chemistry," *Gardeners' Chronicle* for December 7 and 14, 1861.

this new analytical process, the mode of its practical application, and the peculiar, valuable results it enables us to attain.

Dialysis may be defined as analysis effected by liquid diffusion—in other words, the separating of liquid substances from each other by taking advantage of their different rates of diffusibility under particular circumstances. Our knowledge of the laws of liquid diffusion was exceedingly imperfect up to the summer of last year, when Mr. Graham published the results of his elaborate researches on this subject. So much of these results as is necessary in order to understand the principle of dialysis, we will endeavor very briefly to explain.

First. There is a great difference in the diffusibility of different substances in the liquid state, just as there is in the gaseous state. If by means of a pipette we convey a solution of any substance (a salt for instance) to the bottom of a jar of distilled water so as to form a distinct stratum, and then leave the jar undisturbed in a uniform temperature, the dissolved salt will always diffuse into the superincumbent water at a certain rate within a certain time. This rate will vary with the nature of the medium into which diffusion takes place; if, for instance, some other fluid be used instead of water. Briefly expressed, the fact amounts to this—that "different substances in solutions of equal strength diffuse unequally in equal times." (Redwood). For instance, common salt diffuses into water twice as fast as Epsom salt, and this latter twice as fast as gum Arabic. Again, if instead of a single substance we convey a mixed solution of two or three substances to the bottom of the said jar, these substances, notwithstanding their mixture, will still maintain their respective rates of diffusion, the more diffusive body traveling most rapidly and showing itself first and most largely in the upper strata of superincumbent liquid. Hence, what in the case of a single body is mere diffusion, in the case of two or more bodies mixed together is a diffnsive separation of them from each other. Such separation of them will be more or less complete in proportion to the difference between their respective diffusibilities.

Secondly. Between highly diffusive substances on the one hand, and feebly diffusive substances on the other, Mr. Graham has established some important grounds of distinction. The only one, however, which concerns us at present is this—viz., that the former affect the crystalline condition, while the latter are not crystallizable, and have, further, the peculiarity of becoming gelatinous when combined with water. Hence, highly diffusible substances he classes together as "crystalloids," and feebly diffusible ones as "colloids" (from *collin* or gelatine, the type of the class). Among the colloids are hydrated silicic acid, hydrated alumina, and other soluble metallic peroxides, isomorphous with the latter body, together with gelatine, albumen, starch, dextrin and the gums, caramel, vegetable and animal extractive matters.

Now, it is characteristic of the bodies just mentioned that, while they are more or less permeable to crystalloids, they are wholly impermeable to other colloids like themselves which may be in solution. For instance, suppose a layer of firm jelly, or some other colloid of a more convenient nature (such as an animal membrane) to be interposed between water on one side, and a mixed solution of common salt and albumen on the other, it will wholly intercept the albumen, but will allow the salt freely to diffuse through its substance into the water on the opposite side.

It is plain, therefore, that although, as was above shown, simple diffusion into water will partially separate mixed bodies from each other, a far more complete separation will be attained by causing the diffusion to take place into water, not directly, but through an intervening membrane, such as a bladder or sheet of parchment. And this is just what is done in dialysis, which is nothing more than the diffusive separation of crystalloid from colloid bodies through a septum of gelatinous matter, the septum allowing the passage of the one, not of the other. The apparatus needed to conduct this process is the simplest possible. It consists of (1) a basin or deep dish containing three or four inches of pure water; (2) a "dialyser," which is merely some kind of membranous septum secured by a bit of string around a light hoop of sheet gutta-percha, so as to form a vessel like a tambourine. Of all the substances yet used for dialytic septa, the most convenient has been found to be the "parchment-paper" made and sold by Messrs. De la Rue & Co. Care must be taken that it is not porous. The mixed fluid to be dialysed is first poured into the hoop upon the surface of the parchment-paper to the depth of half an inch or so. The dialyser is then floated on the basin or dish of water, into which the crystalloid constituents of the mixture gradually diffuse, the colloid constituents remaining behind. Mr. Graham found that half a litre of urine, dialysed for twenty four hours, gave its crystalloidal constituents to the external water. The latter on evaporation yielded a white saline mass, from which urea was extracted by alcohol in so pure a condition as to appear in crystalline tufts upon the evaporation of the alcohol. Professor Redwood observes that ordinary septa can oly be used in dialysing aqueous solutions; a septum suitable for dialysing alcoholic or ethereal solutions not having yet been discovered. Some form of collodion, he suggests, may possibly answer the purpose.

The process of dialysis admits of some very important practical applications, to which we will briefly allude. (1). It permits of the isolation of various chemical substances in a state of purity in which we were not previously aware of their being able to exist. For instance, chemists had hitherto never succeeded in obtaining a perfectly pure solution of silica. The solution of it, obtained by treating silicate of soda with hydrochloric acid, was not pure; it always contained a certain quantity of hydrochloric acid and chloride of sodium, which resisted all further attempts at separation. But by subjecting the said silica solution to the process of dialysis, the acid and salt, being crystalloids, diffuse out, while the silica, being a colloid, remains behind dissolved in water and perfectly pure. In like manner, dialysis enables us to obtain solutions of peroxide of iron, alumina, and several other bodies, perfectly free from the salts or other chemical agents hitherto indispensable to their solution. (2). In medico-legal inquiries, it affords a most valuable means of separating arsenious acid and the various poisonous metallic salts from their organic solutions. For instance, let a portion of tissue suspected to contain arsenic be chopped into small pieces, soaked in pure water, and then thrown on the dialyser. At the end of twenty-four hours the arsenic, even if its quantity be infinitesimally small, will have diffused into the external water in a state fit for the immediate application of chemical tests. The poison is thus eliminated free from all organic impurity, and without employing any other agent than distilled water—advantages which any one conversant with the usual processes for separating minute quantities of arsenic will not fail to appreciate. Vegetable poisons, such as strychnine, morphine, and the other poisonous alkaloids, may be separated from their organic solutions in precisely the same manner. (3). Professor Redwood suggests its application to another purpose, viz., "the separation of the more active crystallizable constituents of vegetable substances from inert colloidal matter, and the production in this way of a new class of medicines, containing the more active principles of plants, partially purified, and in the state of combination in which they exist in nature." Such preparations would occupy an intermediate place between tinctures, decoctions, and extracts, on the one hand, and the pure, active principles which they often contain (such as alkaloids), on the other. The advantages of vegetable remedies in this form would be greater uniformity of strength, certainty of action, and convenience of administration. They would also keep better, and being void of all inert matter they would be *purely* medicinal, which in their present crude state they are not. The difficulties in the way of their preparation would be great, but probably not insurmountable. (4). It affords a partial explanation of certain points in the physiology of animals and plants hitherto involved in much obscurity. (We say "partial" explanation, because, in all the processes about to be mentioned, a *vital* as well as a *physical* force is at work. At any rate, their full phenomena take place only where life is present; they cannot be imitated outside living organisms). Professor Redwood instances the processes of absorption and secretion accompanying the act of digestion. The mucous membrane of the stomach and intestines may be compared to a dialytic septum between the blood on the one side, and the blood-making constituents of food on the other. Dilute liquids taken into the stomach diffuse through, or (as we generally say) are absorbed by, its mucous membrane. The plastic constituents of food, on the other hand, being colloids, "are retained in the stomach, while the act of digestion proceeds under the influence of crystalloids that are dialysed into that organ, and then pass on to undergo new changes connected with absorption, assimilation and excretion." He further observes that "the action of medicines must be considerably influenced by the state in which they exist as crystalloids or colloids. Thus, iron in the state of chloride, sulphate, or other crystalloidal salt, would be diffused through the walls of the stomach; but not so if in the state of a colloid, such as basic chloride or basic nitrate, in which state it would pass into the intestines, exerting its action probably through the entire length of the alimentary canal." When we know more of the comparative diffusive power of different medicinal preparations than we do at present, we shall probably prescribe them with greater success.

Lastly. Professor Daubeny, of Oxford, has shown, very clearly and fully, how and to what extent the principle of dialysis explains certain phenomena of vegetation—such as the transmission of sap through a plant, the separation of its various secretions from each other, and their maintenance in a state of isolation in appropriate receptacles. (1). The sap is propelled upward through the plant partly by capillarity, partly by atmospheric pressure, owing to the evaporation from the leaves and the partial vacuum thereby occasioned. But it makes its way *into* the plant, in the first instance, by endosmosis through the spongioles of the roots. (2). The particular compounds secreted from the sap in different parts of the plant are maintained in their state of isolation and purity by the same principle of dialysis. The peculiar juices of plants (starch, gum, oils, &c.,) are generally colloids, and therefore have no tendency to pass through the walls of the cells in which they have been elaborated. The different acid and alkaline products, on the other hand, being crystalloids, permeate membrane freely, " but are only temporary constituents or steps in the series of changes which are intended to convert carbonic acid into sugar and starch, and they are consequently got rid of either by exosmosis or else by some other chemical process by which they are converted into glucose or fruit sugar." The principle of dialysis has likewise important bearings on the nature of the ultimate molecules of matter, and on certain geological phenomena. These, however, possess more interest to the geologist and physicist than the physician.—*Medical Times and Gazette.*

